# 5-Chloro-3-cyclo­hexyl­sulfonyl-2-methyl-1-benzofuran

**DOI:** 10.1107/S1600536811008270

**Published:** 2011-03-09

**Authors:** Hong Dae Choi, Pil Ja Seo, Byeng Wha Son, Uk Lee

**Affiliations:** a Department of Chemistry, Dongeui University, San 24 Kaya-dong Busanjin-gu, Busan 614-714, Republic of Korea; b Department of Chemistry, Pukyong National University, 599-1 Daeyeon 3-dong, Nam-gu, Busan 608-737, Republic of Korea

## Abstract

In the title compound, C_15_H_17_ClO_3_S, the cyclo­hexyl ring adopts a chair conformation. In the crystal, mol­ecules are linked through weak inter­molecular C—H⋯O and C—H⋯π inter­actions.

## Related literature

For the pharmacological activity of benzofuran compounds, see: Aslam *et al.* (2006 ▶); Galal *et al.* (2009 ▶); Khan *et al.* (2005 ▶). For natural products with benzofuran rings, see: Akgul & Anil (2003 ▶); Soekamto *et al.* (2003 ▶). For structural studies of related 3-aryl­sulfonyl-5-chloro-2-methyl-1-benzofuran derivatives, see: Choi *et al.* (2008 ▶, 2010 ▶).
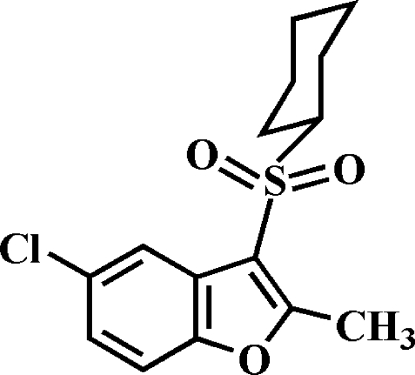


## Experimental

### 

#### Crystal data

C_15_H_17_ClO_3_S
*M* *_r_* = 312.80
Monoclinic, 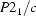

*a* = 14.3135 (2) Å
*b* = 9.2829 (2) Å
*c* = 11.3433 (2) Å
β = 107.566 (1)°
*V* = 1436.91 (4) Å^3^
*Z* = 4
Mo *K*α radiation
μ = 0.42 mm^−1^
*T* = 173 K
0.29 × 0.18 × 0.11 mm

#### Data collection

Bruker SMART APEXII CCD diffractometer
Absorption correction: multi-scan (*SADABS*; Bruker, 2009 ▶) *T*_min_ = 0.891, *T*_max_ = 0.954
13251 measured reflections
3287 independent reflections
2856 reflections with *I* > 2σ(*I*)
*R*_int_ = 0.027

#### Refinement

*R*[*F* ^2^ > 2σ(*F* ^2^)] = 0.034
*wR*(*F* ^2^) = 0.093
*S* = 1.07
3287 reflections
182 parameters
H-atom parameters constrained
Δρ_max_ = 0.49 e Å^−3^
Δρ_min_ = −0.37 e Å^−3^

### 

Data collection: *APEX2* (Bruker, 2009 ▶); cell refinement: *SAINT* (Bruker, 2009 ▶); data reduction: *SAINT*; program(s) used to solve structure: *SHELXS97* (Sheldrick, 2008 ▶); program(s) used to refine structure: *SHELXL97* (Sheldrick, 2008 ▶); molecular graphics: *ORTEP-3* (Farrugia, 1997 ▶) and *DIAMOND* (Brandenburg, 1998 ▶); software used to prepare material for publication: *SHELXL97*.

## Supplementary Material

Crystal structure: contains datablocks global, I. DOI: 10.1107/S1600536811008270/bh2340sup1.cif

Structure factors: contains datablocks I. DOI: 10.1107/S1600536811008270/bh2340Isup2.hkl

Additional supplementary materials:  crystallographic information; 3D view; checkCIF report

## Figures and Tables

**Table 1:** Hydrogen-bond geometry (Å, °) *Cg* is the centroid of the benzene ring.

*D*—H⋯*A*	*D*—H	H⋯*A*	*D*⋯*A*	*D*—H⋯*A*
C6—H6⋯O3^i^	0.95	2.54	3.2630 (19)	133
C10—H10⋯O2^ii^	1.00	2.42	3.3899 (19)	162
C9—H9*C*⋯*Cg*^iii^	0.98	2.69	3.577 (2)	151

Symmetry codes: (i) 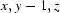; (ii) 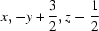; (iii) 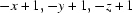.

**Fig. 1.**
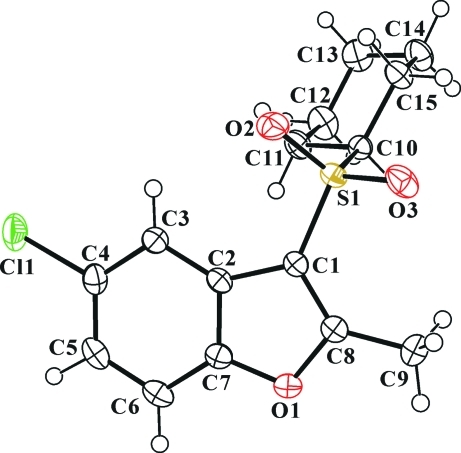
The molecular structure of the title compound. Displacement ellipsoids are drawn at the 50% probability level and H atoms are presented as small spheres of arbitrary radius.

**Fig. 2.**
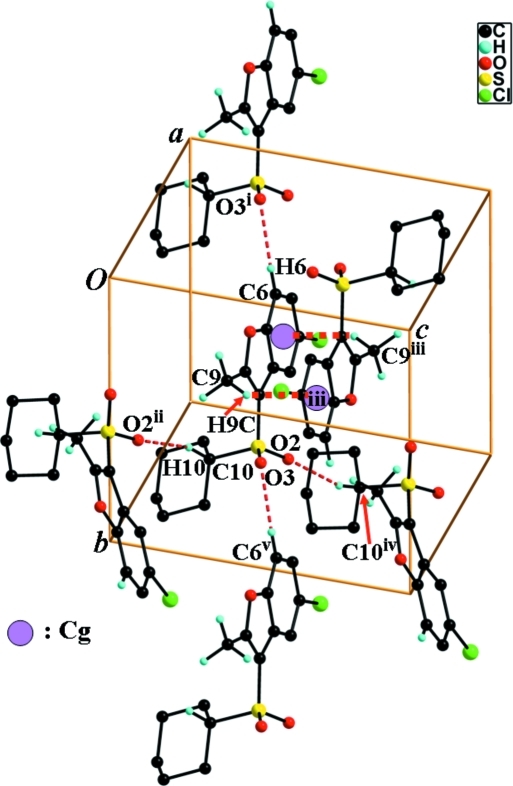
A view of the C–H···O and C–H···π interactions (dotted lines) in the crystal structure of the title compound [Symmetry codes: (i) x, y-1, z; (ii) x, -y+3/2, z-1/2; (iii) -x+1, -y+1, -z+1; (iv) x, -y+3/2, z+1/2; (v) x, y+1, z].

## supplementary crystallographic information

### Comment

Many compounds containing a benzofuran ring exhibit interesting pharmacological properties such as antifungal, antitumor and antiviral, and antimicrobial activities (Aslam *et al.*, 2006; Galal *et al.*, 2009; Khan *et al.*, 2005). These compounds occur in a wide range of natural products (Akgul & Anil, 2003; Soekamto *et al.*, 2003). As a part of our ongoing study of the substituent effect on the solid state structures of 3-arylsulfonyl-5-chloro-2-methyl-1-benzofuran analogues (Choi *et al.*, 2008, 2010), we report herein the crystal structure of the title compound.

In the title molecule (Fig. 1), the benzofuran unit is essentially planar, with a mean deviation of 0.009 (1) Å from the least-squares plane defined by the nine constituent atoms. The cyclohexyl ring is in the chair form. The molecular packing (Fig. 2) is stabilized by weak intermolecular C–H···O hydrogen bonds; the first one between a benzene H atom and the O atom of the sulfonyl unit (Table 1: C6–H6···O3^i^), and the second one between a cyclohexyl H atom and the O atom of the sulfonyl unit (Table 1: C10–H10···O2^ii^). The crystal packing (Fig. 2) is further stabilized by intermolecular C–H···π interactions between a methyl H atoms and the benzene rings (Table 1: C9–H9C···Cg^iii^, Cg is the centroid of the C2···C7 benzene ring).

### Experimental

77% 3-chloroperoxybenzoic acid (515 mg, 2.3 mmol) was added in small portions to a stirred solution of 5-chloro-3-cyclohexylsulfanyl-2-methyl-1-benzofuran (389 mg, 1.1 mmol) in dichloromethane (40 mL) at 273 K. After being stirred at room temperature for 5 h, the mixture was washed with saturated sodium bicarbonate solution and the organic layer was separated, dried over magnesium sulfate, filtered and concentrated at reduced pressure. The residue was purified by column chromatography (hexane-ethyl acetate, 4:1 *v*/*v*) to afford the title compound as a colorless solid [yield 73%, m.p. 440-441 K; *R*_f_ = 0.63 (hexane-ethyl acetate, 4:1 *v*/*v*)]. Single crystals suitable for X-ray diffraction were prepared by slow evaporation of a solution of the title compound in ethyl acetate at room temperature.

### Refinement

All H atoms were positioned geometrically and refined using a riding model, with C–H = 0.95 Å for aryl, 1.00 Å for methine, 0.99 Å for methylene and 0.98 Å for methyl H atoms, respectively. *U*_iso_(H) = 1.2*U*_eq_(C) for aryl, methine and methylene, and 1.5*U*_eq_(C) for methyl H atoms.

### Crystal data

**Table:**

C_15_H_17_ClO_3_S	*F*(000) = 656
*M**_r_* = 312.80	*D*_x_ = 1.446 Mg m^−^^3^
Monoclinic, *P*2_1_/*c*	Melting point: 440 K
Hall symbol: -P 2ybc	Mo *K*α radiation, λ = 0.71073 Å
*a* = 14.3135 (2) Å	Cell parameters from 5109 reflections
*b* = 9.2829 (2) Å	θ = 2.7–27.5°
*c* = 11.3433 (2) Å	µ = 0.42 mm^−^^1^
β = 107.566 (1)°	*T* = 173 K
*V* = 1436.91 (4) Å^3^	Block, colourless
*Z* = 4	0.29 × 0.18 × 0.11 mm

### Data collection

**Table:**

Bruker SMART APEXII CCD diffractometer	3287 independent reflections
Radiation source: rotating anode	2856 reflections with *I* > 2σ(*I*)
graphite multilayer	*R*_int_ = 0.027
Detector resolution: 10.0 pixels mm^-1^	θ_max_ = 27.5°, θ_min_ = 1.5°
φ and ω scans	*h* = −18→18
Absorption correction: multi-scan (*SADABS*; Bruker, 2009)	*k* = −12→10
*T*_min_ = 0.891, *T*_max_ = 0.954	*l* = −14→14
13251 measured reflections	

### Refinement

**Table:**

Refinement on *F*^2^	Primary atom site location: structure-invariant direct methods
Least-squares matrix: full	Secondary atom site location: difference Fourier map
*R*[*F*^2^ > 2σ(*F*^2^)] = 0.034	Hydrogen site location: difference Fourier map
*wR*(*F*^2^) = 0.093	H-atom parameters constrained
*S* = 1.07	*w* = 1/[σ^2^(*F*_o_^2^) + (0.0479*P*)^2^ + 0.5583*P*] where *P* = (*F*_o_^2^ + 2*F*_c_^2^)/3
3287 reflections	(Δ/σ)_max_ = 0.001
182 parameters	Δρ_max_ = 0.49 e Å^−^^3^
0 restraints	Δρ_min_ = −0.36 e Å^−^^3^
0 constraints	

### Fractional atomic coordinates and isotropic or equivalent isotropic displacement parameters (Å^2^)

**Table:**

	*x*	*y*	*z*	*U*_iso_*/*U*_eq_	
S	0.31521 (3)	0.72877 (4)	0.40822 (3)	0.02105 (11)	
Cl	0.18841 (3)	0.20832 (5)	0.64837 (4)	0.03919 (13)	
O2	0.28307 (9)	0.73602 (12)	0.51704 (10)	0.0298 (3)	
O1	0.43749 (7)	0.36827 (12)	0.34930 (10)	0.0255 (2)	
O3	0.38824 (8)	0.82960 (12)	0.39699 (11)	0.0320 (3)	
C1	0.35822 (10)	0.55463 (15)	0.40040 (13)	0.0208 (3)	
C2	0.33118 (10)	0.42681 (15)	0.45538 (13)	0.0209 (3)	
C3	0.27018 (10)	0.39605 (17)	0.52741 (14)	0.0242 (3)	
H3	0.2347	0.4694	0.5537	0.029*	
C4	0.26403 (11)	0.25316 (18)	0.55855 (15)	0.0266 (3)	
C5	0.31494 (11)	0.14251 (17)	0.52184 (15)	0.0294 (3)	
H5	0.3075	0.0459	0.5452	0.035*	
C6	0.37639 (11)	0.17320 (17)	0.45146 (15)	0.0284 (3)	
H6	0.4125	0.1000	0.4260	0.034*	
C7	0.38230 (10)	0.31567 (16)	0.42022 (14)	0.0230 (3)	
C8	0.42122 (10)	0.51346 (16)	0.33784 (13)	0.0226 (3)	
C9	0.47360 (11)	0.59044 (18)	0.26251 (15)	0.0292 (3)	
H9A	0.4262	0.6427	0.1956	0.044*	
H9B	0.5091	0.5208	0.2272	0.044*	
H9C	0.5201	0.6588	0.3148	0.044*	
C10	0.21039 (10)	0.74609 (15)	0.27476 (13)	0.0208 (3)	
H10	0.2316	0.7276	0.1998	0.025*	
C11	0.13094 (11)	0.63719 (17)	0.27601 (15)	0.0285 (3)	
H11A	0.1104	0.6511	0.3511	0.034*	
H11B	0.1570	0.5382	0.2778	0.034*	
C12	0.04281 (12)	0.6570 (2)	0.16061 (17)	0.0362 (4)	
H12A	0.0626	0.6364	0.0859	0.043*	
H12B	−0.0094	0.5880	0.1628	0.043*	
C13	0.00340 (12)	0.8095 (2)	0.15363 (18)	0.0367 (4)	
H13A	−0.0523	0.8209	0.0773	0.044*	
H13B	−0.0209	0.8278	0.2253	0.044*	
C14	0.08265 (12)	0.91795 (19)	0.15367 (16)	0.0338 (4)	
H14A	0.0561	1.0166	0.1522	0.041*	
H14B	0.1027	0.9049	0.0781	0.041*	
C15	0.17212 (11)	0.90049 (16)	0.26764 (15)	0.0274 (3)	
H15A	0.2241	0.9684	0.2627	0.033*	
H15B	0.1539	0.9231	0.3431	0.033*	

### Atomic displacement parameters (Å^2^)

**Table:**

	*U*^11^	*U*^22^	*U*^33^	*U*^12^	*U*^13^	*U*^23^
S	0.02279 (18)	0.01684 (18)	0.02228 (19)	0.00067 (12)	0.00493 (14)	−0.00147 (13)
Cl	0.0382 (2)	0.0426 (3)	0.0397 (3)	−0.00341 (17)	0.01619 (19)	0.01558 (19)
O2	0.0390 (6)	0.0287 (6)	0.0215 (5)	0.0062 (5)	0.0088 (5)	−0.0033 (4)
O1	0.0275 (5)	0.0225 (5)	0.0282 (6)	0.0036 (4)	0.0111 (4)	−0.0011 (4)
O3	0.0265 (5)	0.0208 (5)	0.0445 (7)	−0.0038 (4)	0.0044 (5)	0.0007 (5)
C1	0.0219 (6)	0.0188 (7)	0.0213 (7)	0.0000 (5)	0.0057 (5)	0.0003 (5)
C2	0.0213 (6)	0.0183 (7)	0.0206 (7)	0.0006 (5)	0.0027 (5)	0.0004 (5)
C3	0.0239 (7)	0.0243 (7)	0.0241 (7)	0.0019 (6)	0.0068 (6)	0.0015 (6)
C4	0.0235 (7)	0.0300 (8)	0.0246 (7)	−0.0025 (6)	0.0047 (6)	0.0063 (6)
C5	0.0329 (8)	0.0203 (7)	0.0302 (8)	−0.0011 (6)	0.0025 (6)	0.0050 (6)
C6	0.0305 (8)	0.0197 (7)	0.0318 (8)	0.0037 (6)	0.0045 (6)	−0.0012 (6)
C7	0.0226 (7)	0.0221 (7)	0.0227 (7)	0.0011 (5)	0.0045 (5)	−0.0011 (6)
C8	0.0220 (6)	0.0221 (7)	0.0220 (7)	−0.0001 (5)	0.0039 (5)	−0.0005 (6)
C9	0.0282 (7)	0.0339 (9)	0.0274 (8)	−0.0029 (6)	0.0114 (6)	0.0012 (7)
C10	0.0214 (7)	0.0206 (7)	0.0196 (7)	0.0007 (5)	0.0052 (5)	0.0002 (5)
C11	0.0274 (7)	0.0225 (7)	0.0326 (8)	−0.0035 (6)	0.0047 (6)	0.0011 (6)
C12	0.0282 (8)	0.0351 (9)	0.0387 (10)	−0.0083 (7)	0.0000 (7)	−0.0002 (8)
C13	0.0231 (8)	0.0417 (10)	0.0403 (10)	0.0006 (7)	0.0022 (7)	0.0053 (8)
C14	0.0286 (8)	0.0321 (9)	0.0363 (9)	0.0039 (6)	0.0030 (7)	0.0096 (7)
C15	0.0264 (7)	0.0211 (7)	0.0319 (8)	0.0014 (6)	0.0046 (6)	0.0030 (6)

### Geometric parameters (Å, °)

**Table:**

S—O3	1.4372 (11)	C9—H9B	0.9800
S—O2	1.4431 (12)	C9—H9C	0.9800
S—C1	1.7416 (15)	C10—C11	1.525 (2)
S—C10	1.7882 (14)	C10—C15	1.528 (2)
Cl—C4	1.7461 (16)	C10—H10	1.0000
O1—C8	1.3673 (18)	C11—C12	1.530 (2)
O1—C7	1.3760 (18)	C11—H11A	0.9900
C1—C8	1.360 (2)	C11—H11B	0.9900
C1—C2	1.447 (2)	C12—C13	1.517 (3)
C2—C7	1.391 (2)	C12—H12A	0.9900
C2—C3	1.393 (2)	C12—H12B	0.9900
C3—C4	1.382 (2)	C13—C14	1.516 (2)
C3—H3	0.9500	C13—H13A	0.9900
C4—C5	1.394 (2)	C13—H13B	0.9900
C5—C6	1.384 (2)	C14—C15	1.529 (2)
C5—H5	0.9500	C14—H14A	0.9900
C6—C7	1.378 (2)	C14—H14B	0.9900
C6—H6	0.9500	C15—H15A	0.9900
C8—C9	1.480 (2)	C15—H15B	0.9900
C9—H9A	0.9800		
			
O3—S—O2	118.27 (7)	C11—C10—C15	111.46 (12)
O3—S—C1	108.80 (7)	C11—C10—S	111.72 (10)
O2—S—C1	107.20 (7)	C15—C10—S	108.96 (10)
O3—S—C10	108.26 (7)	C11—C10—H10	108.2
O2—S—C10	108.50 (7)	C15—C10—H10	108.2
C1—S—C10	105.04 (7)	S—C10—H10	108.2
C8—O1—C7	107.10 (11)	C10—C11—C12	109.68 (13)
C8—C1—C2	107.48 (13)	C10—C11—H11A	109.7
C8—C1—S	126.00 (11)	C12—C11—H11A	109.7
C2—C1—S	126.49 (11)	C10—C11—H11B	109.7
C7—C2—C3	119.53 (13)	C12—C11—H11B	109.7
C7—C2—C1	104.62 (13)	H11A—C11—H11B	108.2
C3—C2—C1	135.84 (13)	C13—C12—C11	110.76 (14)
C4—C3—C2	116.49 (14)	C13—C12—H12A	109.5
C4—C3—H3	121.8	C11—C12—H12A	109.5
C2—C3—H3	121.8	C13—C12—H12B	109.5
C3—C4—C5	123.47 (15)	C11—C12—H12B	109.5
C3—C4—Cl	118.45 (13)	H12A—C12—H12B	108.1
C5—C4—Cl	118.08 (12)	C14—C13—C12	110.68 (14)
C6—C5—C4	120.08 (15)	C14—C13—H13A	109.5
C6—C5—H5	120.0	C12—C13—H13A	109.5
C4—C5—H5	120.0	C14—C13—H13B	109.5
C7—C6—C5	116.40 (14)	C12—C13—H13B	109.5
C7—C6—H6	121.8	H13A—C13—H13B	108.1
C5—C6—H6	121.8	C13—C14—C15	111.43 (14)
O1—C7—C6	125.55 (14)	C13—C14—H14A	109.3
O1—C7—C2	110.42 (13)	C15—C14—H14A	109.3
C6—C7—C2	124.03 (14)	C13—C14—H14B	109.3
C1—C8—O1	110.38 (13)	C15—C14—H14B	109.3
C1—C8—C9	134.19 (14)	H14A—C14—H14B	108.0
O1—C8—C9	115.43 (13)	C10—C15—C14	109.85 (13)
C8—C9—H9A	109.5	C10—C15—H15A	109.7
C8—C9—H9B	109.5	C14—C15—H15A	109.7
H9A—C9—H9B	109.5	C10—C15—H15B	109.7
C8—C9—H9C	109.5	C14—C15—H15B	109.7
H9A—C9—H9C	109.5	H15A—C15—H15B	108.2
H9B—C9—H9C	109.5		
			
O3—S—C1—C8	−29.39 (15)	C3—C2—C7—C6	0.5 (2)
O2—S—C1—C8	−158.38 (13)	C1—C2—C7—C6	−178.90 (14)
C10—S—C1—C8	86.33 (14)	C2—C1—C8—O1	−0.51 (16)
O3—S—C1—C2	152.86 (12)	S—C1—C8—O1	−178.61 (10)
O2—S—C1—C2	23.87 (14)	C2—C1—C8—C9	179.31 (15)
C10—S—C1—C2	−91.41 (13)	S—C1—C8—C9	1.2 (3)
C8—C1—C2—C7	0.17 (16)	C7—O1—C8—C1	0.64 (16)
S—C1—C2—C7	178.26 (11)	C7—O1—C8—C9	−179.21 (12)
C8—C1—C2—C3	−179.09 (16)	O3—S—C10—C11	177.11 (11)
S—C1—C2—C3	−1.0 (2)	O2—S—C10—C11	−53.37 (12)
C7—C2—C3—C4	−0.6 (2)	C1—S—C10—C11	61.01 (12)
C1—C2—C3—C4	178.57 (15)	O3—S—C10—C15	−59.29 (12)
C2—C3—C4—C5	0.0 (2)	O2—S—C10—C15	70.23 (12)
C2—C3—C4—Cl	−179.83 (11)	C1—S—C10—C15	−175.40 (10)
C3—C4—C5—C6	0.7 (2)	C15—C10—C11—C12	57.47 (17)
Cl—C4—C5—C6	−179.44 (12)	S—C10—C11—C12	179.64 (12)
C4—C5—C6—C7	−0.8 (2)	C10—C11—C12—C13	−57.59 (19)
C8—O1—C7—C6	178.58 (14)	C11—C12—C13—C14	57.6 (2)
C8—O1—C7—C2	−0.52 (16)	C12—C13—C14—C15	−56.9 (2)
C5—C6—C7—O1	−178.78 (14)	C11—C10—C15—C14	−56.53 (17)
C5—C6—C7—C2	0.2 (2)	S—C10—C15—C14	179.73 (11)
C3—C2—C7—O1	179.63 (12)	C13—C14—C15—C10	55.92 (19)
C1—C2—C7—O1	0.21 (16)		

### Hydrogen-bond geometry (Å, °)

**Table:**

Cg is the centroid of the benzene ring.

**Table:**

*D*—H···*A*	*D*—H	H···*A*	*D*···*A*	*D*—H···*A*
C6—H6···O3^i^	0.95	2.54	3.2630 (19)	133
C10—H10···O2^ii^	1.00	2.42	3.3899 (19)	162
C9—H9C···Cg^iii^	0.98	2.69	3.577 (2)	151

Symmetry codes: (i) *x*, *y*−1, *z*; (ii) *x*, −*y*+3/2, *z*−1/2; (iii) −*x*+1, −*y*+1, −*z*+1.
